# The Effectiveness of Drinking and Driving Policies for Different Alcohol-Related Fatalities: A Quantile Regression Analysis

**DOI:** 10.3390/ijerph10104628

**Published:** 2013-09-27

**Authors:** Yung-Hsiang Ying, Chin-Chih Wu, Koyin Chang

**Affiliations:** 1 College of Management, National Taiwan Normal University, Taipei, 106, Taiwan; E-Mail: yying@ntnu.edu.tw; 2 Institute of China and Asia-Pacific Studies, National Sun Yat-sen University, 70 Lienhai Rd., Kaohsiung 804, Taiwan; 3 Department of Healthcare Information and Management, Ming Chuan University, 250 Chung-Shan N. Rd., Taipei 111, Taiwan

**Keywords:** quantile regression analysis, alcohol-related traffic fatalities, policies

## Abstract

To understand the impact of drinking and driving laws on drinking and driving fatality rates, this study explored the different effects these laws have on areas with varying severity rates for drinking and driving. Unlike previous studies, this study employed quantile regression analysis. Empirical results showed that policies based on local conditions must be used to effectively reduce drinking and driving fatality rates; that is, different measures should be adopted to target the specific conditions in various regions. For areas with low fatality rates (low quantiles), people’s habits and attitudes toward alcohol should be emphasized instead of transportation safety laws because “preemptive regulations” are more effective. For areas with high fatality rates (or high quantiles), “*ex-post* regulations” are more effective, and impact these areas approximately 0.01% to 0.05% more than they do areas with low fatality rates.

## 1. Introduction

Driving under the influence of alcohol has long been a severe social problem in the United States. In 2009, a study by the National Highway Traffic Safety Administration (NHTSA) indicated that approximately 30 people died in alcohol-related collisions per day (approximately 11,000 deaths per year); that is, one person dies in an alcohol-related collision every 48 min. Additionally, this horrifying figure was the result of already improved traffic safety conditions (the data provided by the NHTSA showed that in approximately 1982, nearly 30,000 people died in alcohol-related collisions in the U.S. per year, which accounted for 60% of the overall traffic crashes. Today that percentage has dropped to 38%). In 1980, Mothers Against Drunk Driving (MADD) was founded in the U.S., dedicating itself to urging state and federal governments to enact a series of drinking and driving policies that significantly reduced alcohol-related fatalities in the U.S. Since then, government officials and scholars have conducted numerous investigations and studies on the effectiveness of drinking and driving policies in reducing alcohol-related fatalities.

The data used in the studies on drunk driving consist of three categories: Cross-sectional data (e.g., Beck *et al*., [1]; Paschall, [2]; Phelps, [3]), time-series data (e.g., Whetten-Goldstein *et al*., [4]; Villaveces *et al*., [5]), and panel data (e.g., Chang *et al*., [6]; Lovenheim and Slemrod, [7]; Hingson *et al*., [8]; Ruhm, [9]; Males, [10]; Cook & Tauchen, [11]; Saffer and Grossman, [12]). Two estimation methods were used in these traditional econometric studies: (1) the ordinary least square (OLS) method that estimates the conditional mean function of dependent variables; and (2) the least absolute deviation (LAD) method that estimates the conditional median function of dependent variables. These two estimation methods emphasize the central tendency distribution of dependent variables and they both address the data at a macro or comprehensive level instead of examining individual quantiles. However, an observation of the alcohol-related fatality data show that we must study the development tendency of the alcohol-related fatalities of individual quantiles in addition to the central tendency development of alcohol-related fatalities. The reasons are as follows:

### 1.1. High Consistency of the U.S. Alcohol-Related Fatalities

**Figure 1:**
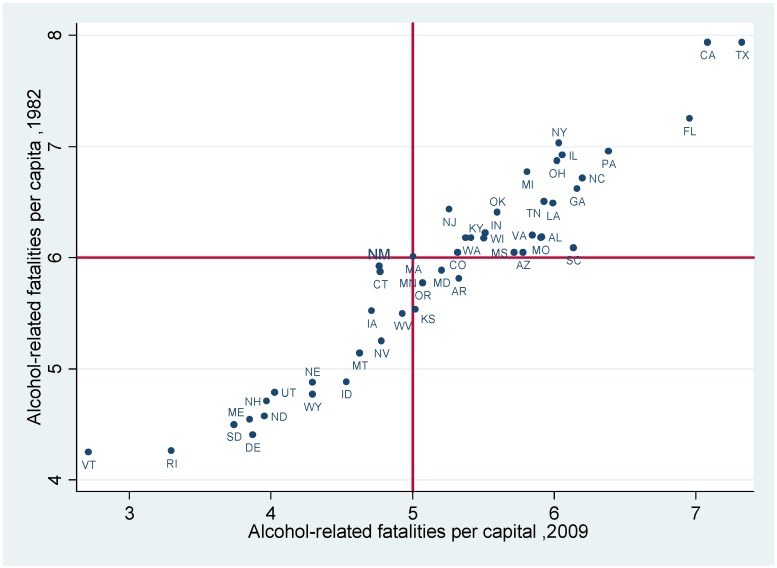
Alcohol-related fatalities in 2009 (horizontal axis) and 1982 (vertical axis).

Figure 1 shows that although U.S. alcohol-related fatalities have declined significantly, the states with high rates of alcohol-related fatalities in 1982 had maintained comparatively high levels in 2009 (e.g., CA, TX, and FL); the opposite situation was also true (e.g., in UT, VT, and RI). Based on this phenomenon, we suspect that drinking and driving policies that showed mean effectiveness had different effects for varying quantiles or alcohol-related fatality rates, preventing the values for states in Quadrant 3 (*i.e.*, the states that maintained high rates of alcohol-related fatalities) from moving toward Quadrant 1 (*i.e.*, the states that had shown high alcohol-related fatalities transformed into states with low alcohol-related fatalities).

### 1.2. Regional Difference in United States Alcohol-Related Fatalities

**Figure 2:**
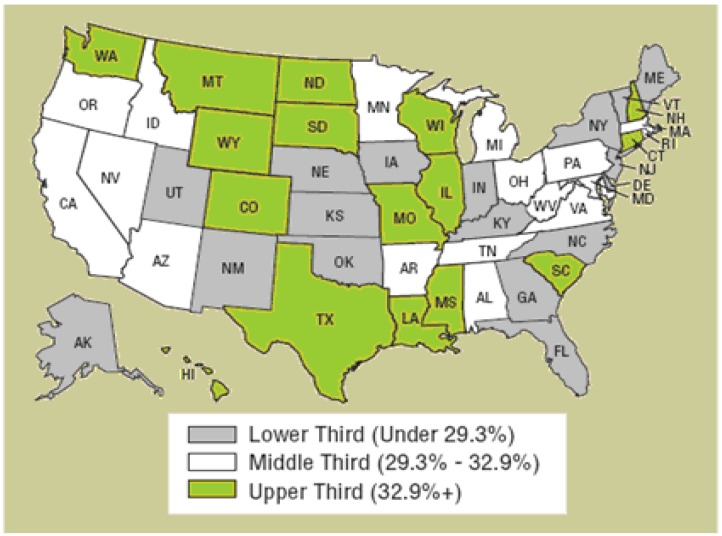
Fatalities as a percentage of total fatalities in crashes involving at least one driver with a BAC=0.08+, 2006 (Source: NHTSA).

Figure 2 shows that the states with relatively high alcohol-related fatalities are situated in the west and the south, whereas the states with relatively low alcohol-related fatalities are situated in the northeast, indicating that U.S. alcohol-related fatality are regional. Chang *et al*. [6] indicated that the drinking and driving policies in different regions had varying effects (In Chang *et al*. [6], the U.S. was divided into Far West, Great Lakes, Mid East, New England, Plains, Rocky Mts., Southeast, and Southwest). We concluded that different drinking and driving policies had different effects depending on the level of alcohol-related fatalities.

This finding provided strong motivation to examine the effectiveness of drinking and driving policies under different alcohol-related fatality rates. To effectively discuss the effects of various drinking and driving policies on alcohol-related fatalities in different quantiles, we used the quantile regression method proposed by Koenker and Bassett [13] for estimation. The simple concept of the advantage of quantile regression, relative to the ordinary least squares regression, is that the quantile regression estimates are more robust against outliers in the response measurements [14]. Other advantages of the quantile regression method include that it makes no distribution assumptions on the population; it supplements the insufficiency of the traditional regression methods, which focuses only on the mean value of alcohol-related fatalities to estimate and interpret drinking and driving policy parameters; and finally, in our study, it specifically identifies the differing effect levels of drinking and driving policies on alcohol-related fatalities in different quantiles. Thus, we believe that applying QR model has advantages over the traditional method.

The structure of this study is as follows: research motivation and objectives are introduced in Section 1. In Section 2, we provide background information for the alcohol control- and road safety-related policies and review empirical studies on drunk driving. In Section 3, we explain the methodology and the quantile regression model. In Section 4, we provide the results of the empirical study; we follow the steps provided in the research methodology to implement empirical research and introduce, interpret, and analyze the results from the conducted empirical study. Finally, in Section 5, we present a conclusion in which the empirical results are integrated and conclusions and suggestions for future studies are provided.

## 2. Background and Literature Review

The establishment of MADD was significant in the history of U.S. drinking and driving policies. Although the first U.S. law against drunk driving was passed in New York in 1910, other state governments and the federal governments did not pass such laws until MADD was founded in 1980, when the organization launched a wave of lobbying and campaigns. This led to a gradual trend toward more complete U.S. drinking and driving laws and policies.

### 2.1. Minimum Legal Drinking Age

In 1933, after the U.S. prohibition of the manufacture and sale of alcoholic beverages was lifted, the states began to set minimum legal drinking ages (MLDA), most of which were 21 years of age. By the early 1970s, most states had lowered their MLDAs to between 18 and 20 years of age, resulting in numerous discussions and studies. Most of these studies showed that the rise and decline of teenage car crash fatalities were related to MLDA [15]. Therefore, in 1984, the U.S. Congress enacted legislation that set the MLDA, stipulating that states that failed to raise their MLDA to 21 would lose a portion of their federal highway construction funding. By 1988, all states had raised their MLDA to 21. MLDA has remained one of the most researched alcohol prevention policies. The studies by Saffer and Grossman [12], Wilkinson [16], Wagenaar [17], Dee [18], Voas *et al*. [19] and Fell *et al*. [20] indicated that raised MLDAs effectively reduced alcohol-related traffic collisions.

### 2.2. Blood Alcohol Concentration

In 1939, the State of Indiana first enacted a blood alcohol concentration (BAC) limit of less than 0.15. In 1983, Oregon and Utah lowered their BAC from 0.1 to 0.08. In a report to Congress in 1991, the NHTSA proposed lowering the BAC to 0.08, and the law limiting BAC was passed by Congress in the same year. In 1998, Congress established the National Mobile Incentive Grant Scheme to strictly enforce the BAC. In 2000, Congress encouraged states to implement BAC restrictions, stipulating that the states that failed to lower their BAC to 0.08 would lose a portion of their federal highway construction funding. By 2004, all states enacted a BAC limit of 0.08.

Hingson *et al*. [21], Fell and Voas [22], Tippetts *et al*. [23], Kaplan and Prato [24], and Wagenaar *et al*. [25] showed that lowering the BAC from 0.10 to 0.08 reduced alcohol-related fatalities by 5% to 16%, saving approximately 400 lives per year.

### 2.3. Zero Tolerance

Zero tolerance was a combination of MLDA and BAC. This act stipulated that drivers under the age of 21 should not demonstrate a BAC exceeding 0.02%. Maryland first passed the Zero Tolerance Law in 1990. In 1995, to encourage other states to enact the Zero Tolerance Law, Congress stipulated under the National Highway Systems Designation Act (NHSDA) that the states that failed to enact the Zero Tolerance Law would lose a portion of their federal highway construction funding. By 1998, all states had implemented the Zero Tolerance Law. Zwerling and Jones [26], Wagenaar *et al*. [27], Voas *et al*. [28], Carpenter *et al*. [29], and Liang and Huang [30] showed that the Zero Tolerance Act reduced alcohol-related fatalities by 4% to 24%.

### 2.4. Open Container Laws

The Open Container Laws regarding drinking and driving stipulated that the drivers would be fined if open containers of alcoholic beverages were found in the cabins of their vehicles. Because this was an interstate law instead of a federal law, the states had the right to decide whether they issued the law and they could also adjust the contents of this law. In 1988, to encourage states to pass the Open Contain Laws, Congress stipulated that states that failed to implement the Open Container Laws would lose a portion of their federal highway construction funding. Currently, only 43 states have enacted this law. Eisenberg [31] and Benson *et al*. [3] showed that this law had a negative correlation with alcohol-related fatalities.

### 2.5. Driving Under the Influence

The Driving Under the Influence (DUI) Law was constructed in the framework of BAC limits. The “Administrative License Revocation” (ALR) and DUI fine were articles of the DUI Law. Under the ALR law, licenses are immediately revoked whenever a driver either: (1) refuses to submit to BAC testing; or (2) submits to testing with results indicating a BAC over the legal limit of 0.08% (by 2011, 42 states had implemented the ALR, leaving eight states not yet adopting the law: Kentucky, Michigan, Montana, New Jersey, Pennsylvania, Rhode Island, South Dakota, and Tennessee). Ruhm [9], Voas *et al*. [32], and Wagenaar and Maldonado-Molina [33] indicated that ALR had significant effects on reducing alcohol-related fatalities. The DUI fines varied among states, with the lowest fines for first-time offenders ranging from US$150 in Wisconsin to US$2,000 in Texas. The results of studies on DUI fines differed. Chaloupka *et al*. [34] and Wagenaar *et al*. [25] indicated that DUI fins had significant effects on reducing alcohol-related fatalities, whereas Sloan *et al.* [35] sowed that DUI fines had no significant effects on reducing alcohol-related fatalities, and Young and Likens [36] found a positive correlation between DUI fines and alcohol-related fatalities.

Based on the definition proposed by Becker and Posner [37], we classifed these drinking and driving policies into two categories: preventive and ex-postregulations. Preventive regulations were enacted to prevent drinking and driving, including the Beer tax, MLDA, and Open Container Laws, whereas ex-post regulations were enacted to penalize drivers under the influence of alcohol, including the 0.08 BAC limit, ALR, the Safety Belt Law, the Zero Tolerance Law, speed limits, and DUI fines. Although some laws such as the beer tax, speed limits, and the Safety Belt Law were not intended to reduce alcohol-related collisions, numerous studies have observed that these laws had direct and significant effects on alcohol-related fatalities. Specifically, the effects of the beer tax on alcohol-related fatalities were widely examined. For example, the empirical results of Chaloupka and Wechsler [38], Phelps [39], Kenkel [40], Saffer and Grossman [12], and Mann *et al*. [41] showed a significant negative correlation between the beer tax and alcohol-related fatalities, whereas the empirical results of Sloan and Githens [42], Dee [18], Mast *et al*. [43], and Young and Likens [36] indicated that the relationship between the beer tax and alcohol-related fatalities was neither significant nor necessarily negatively correlated.

## 3. Research Model and Methodology

### 3.1. Panel Data Quantile Regression Model

The quantile regression (QR) analysis was proposed in Koenker and Bassett [13] as an expansion of the least absolute deviation (LAD). QR can be used to detail the performance of explanatory variables under the influence of conditional medians. Additionally, it can be expanded to analyze the performance of variables under the influence of different conditional quantiles.

Based on the descriptions in the study by Koenker & Bassett (1978), we established a random variable cumulative distribution function, as shown in Equation (1):

Py(*y_it_ < y*│*x_it_*) *= F*(*y − x_it_β│x_it_*) = *τ*, τ ∈ (0,1) (1)

where *y_it_* represents the dependent explanatory variable vector, and *x_it_* is the independent explanatory variable vector. *β* is the regression coefficient vector obtained through estimation satisfying (1) and varies according to different quantiles *τ*. Therefore, *β*(*τ*) represents the regression coefficient vector under the influence of the τth quartile.

We simplified (1) into a basic panel data quantile regression model, as shown in Equation (1.1). (Equation (1) can be simplified into the conditional quantile form: 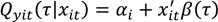, where *Q_yit_*(*τ*│*x_it_*) represents the conditional quantile of *yit* under a set, xit assuming *Q_yit_*(*ε_it_*(*τ*)│*x_it_*) = 0):

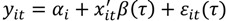
(1.1)
where *ε_it_*(*τ*) represents the random error under quantile *τ*, and *α_i_* represents the regional fixed effects that are unaffected by quantile (*τ*) and capture unobserved time-invariant heterogeneity between regions [14]. Also included is the state-specific time fixed effect to guarantee that the results are not due to the trend of fatalities caused by drunk driving [9]. The conditional expectation value in traditional panel data analysis is a linear operator; thus, within group estimation is used to eliminate the *α_i_* in the model and prevent biased estimation. However, the conditional quantile in the QR analysis is not a linear estimator, and within group estimation cannot be used to eliminate the fixed effects. Therefore, Koenker [14] introduced an objective function with penalty terms to eliminate the fixed effects, as shown in Equation (2):

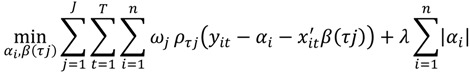
(2)
where 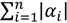 is the penalty. When *λ* = 0, it represents the traditional fixed effects, and when *λ* > 0, it represents the fixed effects with a penalty. Thus, the panel data QR estimated value 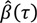 under fixed effects can be obtained [14] verified that 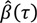 was the consistency estimation equation for *β*(*τ*) and its progressive distribution was normal distribution] representing the marginal effects of different quantile explanatory variables on the explained variables when other explanatory variables *x_i_* were controlled. In other words, when *x_i_* changes by one unit, the quantile *τ* value of the explained variable changes by 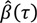 units.

Based on the suggestions in Lamarche [44], we used the bootstrap method for sampling estimation. In this method, the re-sampling of samples was used to simulate the population distribution. We also relaxed the assumption limit that requires the conditional distribution of the errors to be homoscedastic [45]. Therefore, a variance matrix estimation equation with consistency was obtained, as shown in Equation (3).

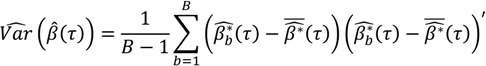
(3)
where 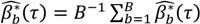.

The QR model can describe the performances of different quantile conditional distributions and therefore can more fully describe the characteristics of samples. This is different from the OLS model describes only the mean marginal effects of the explanatory variables on the explained variables.

### 3.2. Empirical Model

Because this model was comparatively suitable, we used the panel data QR model to explore and verify whether changes in the effectiveness of drinking and driving policies occur with varying levels of alcohol-related fatalities. Based on the framework in Koenker [14], we established an empirical model for panel data QR, as shown in Equation (4):

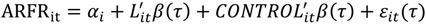
(4)
where *ε_it_*(*τ*)and *α_i_* are explained in the paragraph following Equation (1.1). Annual data from the 48 contiguous states for the years 1982 to 2009 are employed. *ARFR_it_* represents the alcohol-related fatalities per 100,000 population (according to Chang *et al*. [6], a lowered ARFR indicates that the traffic conditions in a state were undergoing improvement, that is, improved traffic conditions were beneficial to reducing alcohol-related fatalities) obtained from the Fatal Accident Reporting System (FARS) of the NHTSA. *CONTROL_it_* represents geo-economic factors, such as population density (Pop. density*_it_*), income (Income*_it_*), unemployment rates (Unemp. rate*_it_*), teenage/young driver ratio (Under24*_it_*), and U.S. administrative districts. *L_it_* represents the nine drinking and driving policies selected for this discussion: The beer tax (Beer tax*_it_*), MLDA (MLDA*_it_*), BAC (Bac08*_it_*), ALR (ALR*_it_*), the Safety Belt Law (Belt*_it_*), the Zero Tolerance Law (Zero tolerance*_it_*), Open Container Laws (Open container*_it_*), the speed limit (Speed limit*_it_*), and DUI fines (DUI fine*_it_*) (these policies have been passed and implemented in all states at different times). These policies were set as the dummy variables in this model except for the beer tax. If states had adopted a policy, it was marked as 1; if they had not, it was marked as 0. Please refer to Table 1 for the details of the variables.

**Table 1:** Variable definition and statistics.

Variable	Definition, mean, SD	Source
**ARFR**	Alcohol-related deaths (BAC 0.1+) resulting from motor vehicle crashes per 100,000 population, mean = 8.23, SD = 3.67	NHTSA
**Income**	Per capita personal income divided by CPI, expressed in thousands of dollars, mean = 24.06, SD = 9.32	Statistical Abstract of the U.S.
**Unemp. rate**	State unemployment rate, mean = 5.76, SD = 2.05	Bureau of Labor Statistics
**Pop. density**	Population per square mile of land area, mean = 4.42, SD = 1.30	Statistical Abstract of the U.S.
**Under24**	Fraction of licensed drivers age 16 to 24 years (Number of licensed drivers age 16 to 24 years as a fraction of total licensed drivers of all ages), mean = 0.16, SD = 0.14	Highway Statistics
**Beer tax**	Sum of Federal and State excise taxes on a case of 24 × 12 oz cans of beer divided by CPI (1982 = 1), mean = 0.4921, SD = 0.04	Brewers’Almanac, U.S. Brewers Association and Significant Features of Fiscal Federalism
**Belt**	Dichotomous variable that is coded as 1 if the state had passed the safety belt law, mean = 0.76, SD = 0.43	NHTSA
**ALR**	Dichotomous variable that is coded as 1 if the state suspends the drivers’ licenses of individuals who are arrested for driving while intoxicated (DWI), mean = 0.64, SD = 0.48	NHTSA
**Bac08**	Dichotomous variable that was coded as 1 if the state considers it an offense to operate a motor vehicle with a BAC at or above 0.08%, mean = 0.38, SD = 0.49	NHTSA
**Zero tolerance**	Dichotomous variable that was coded as 1 if the state made it illegal *per se* for persons under the age of 21 to drive with any measurable amount of alcohol in their blood, mean = 0.53, SD = 0.49	NHTSA
**MLDA**	Minimum legal drinking age in years for the purchase and consumption of beer, alcoholic content more than 3.2%, mean = 0.91, SD = 0.29	NHTSA
**Speed limit**	Dichotomous variable that was coded as 1 if the state mandated a maximum speed limit of 70 mph for its rural state highways, mean = 0.91, SD = 0.45	Insurance Institute for Highway Safety
**Open container**	Dichotomous variable that was coded as 1 if the state prohibited possessing and/or drinking from an open container of alcohol in moving motor vehicles in certain areas, mean = 0.25, SD = 0.43	Alcohol policy information system (APIS)
**DUI fine**	Dichotomous variable that was coded as 1 if the state passed DUI fine laws, mean = 0.53, SD = 0.50	Each State Government
**Northwest**	States include CT, MA, ME, NH, NY, PA, RI, VT	U.S. Bureau of Economic Analysis
**Midwest**	States include IA, IL, IN, KS, MN, MO, MI, ND, NE, OH, SD, WI	
**West**	States include AZ, CA, CO, ID, MT, NM, NV, OR, UT, WA, WY	
**South**	States include AL, AR DC, DE, FL, GA, KY, LA, MD, MS, NC, OK, SC, TN, TX, VA, WV	

Note: 1. NHTSA represents National Highway Traffic Safety Administration; 2. The abbreviation of each state in the USA are explained in Table A1.

## 4. Results

To clearly explain the estimation results of QR with different quantiles, we use the definition of QR 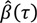 to classify alcohol-related fatalities into three types based on their quantiles: (1) *τ* = 0.25 represented that the area had a low rate of alcohol-related fatalities; (2) *τ* = 0.5 represented that the area had a medium rate of alcohol-related fatalities; and (3) *τ* = 0.75 represented that the area had a high rate of alcohol-related fatalities. We then conducted empirical analyses based on Equation (4) to discuss the effects of drinking and driving policies and other control variables on alcohol-related fatalities.

Table 2 shows four characteristics: (1) In the areas with low rates of alcohol-related fatalities, increases in unemployment rates and the number of young drivers (licensed drivers aged between 16 and 24 years of age) correlated with significant increases in alcohol-related fatalities. In these areas, preventive regulations (such as MLDA and the beer tax) were relatively more effective in reducing alcohol-related fatalities than ex-post regulations; (2) In areas with high rates of alcohol-related fatalities, socio-economic factors such as employment rate, and the number of young drivers had no significant effects on fatalities. In these areas, ex-post regulations (such as BAC limit (0.08) and ALR) correlated with reductions in fatalities at 1% significance level; (3) In terms of regional fixed effect, all coefficients of three regions are negative, indicating that the omitted region, South, had the highest alcohol-related fatalities rate. Since the second highest region was West, our results appear to support the original finding described in Figure 2; (4) The effects of preventive regulations declined as the rate of alcohol-related fatalities increased, whereas the opposite was observed for ex-post regulations. This indicates that in areas with high rates of alcohol-related fatalities, ex-post regulations were more effective than preventive regulations. The only ineffective traffic law in reducing alcohol-related fatalities in all quantiles is the speed limit. In the following section, we detail the effectiveness of various drinking and driving policies and other control variables in areas with high, medium, and low rates of alcohol-related fatalities.

### 4.1. Areas with Low Alcohol-Related Fatalities

Table 2 shows that all drinking and driving polices except for speed limit had significant effects on lowering rates of alcohol-related fatalities in these areas. Among all policies, the beer tax was the most effective in lowering fatalities. Assuming that other conditions remained constant, when the beer tax increased by 1%, the rate of alcohol-related fatalities declined by 0.41%. Additionally, zero tolerance, the Open Container Law, and BAC effectively reduced the rate of fatalities in these areas, showing decreases of 0.18%, 0.14%, and 0.06%, respectively.

Other economic and demographic variables, such as per capita income, unemployment rates, and the number of young drivers all had significant effects in these areas at 5% level. Unemployment rates and the number of young drivers have a significant positive correlation with alcohol-related fatalities, that is, increases in unemployment rates and the proportion of young drivers caused an increase in fatalities. In particular, when the number of young drivers increased 1%, the rate of fatalities increased 0.08% holding other conditions constant. Conversely, per capita income had a significant negative correlation with alcohol-related fatalities. Assuming that other conditions remained constant, when the per capita income increased 1%, the alcohol-related fatalities declined 0.036%.

**Table 2:** Panel data quantile regression analysis.

Variable	25 percentile		50 percentile		75 percentile	
(ARFR)	Coeff	*Z*-value	Coeff	*Z*-value	Coeff	*Z*-value
**Income**	−0.036	0.020 **	−0.016	0.072 *	−0.026	0.018 **
**Unemp. rate**	0.031	0.000 ***	0.011	0.066 *	0.004	0.548
**Pop. Density**	−0.166	0.000 ***	−0.165	0.000 ***	−0.154	0.000 ***
**Under24**	0.081	0.000 ***	0.042	0.236	0.006	0.882
**Beer tax**	−0.413	0.000 ***	−0.312	0.000 ***	−0.252	0.031 **
**Belt**	−0.051	0.041 **	−0.064	0.004 ***	−0.042	0.011 **
**ALR**	−0.058	0.025 **	−0.054	0.034 **	−0.065	0.000 ***
**Bac08**	−0.066	0.002 ***	−0.072	0.000 ***	−0.101	0.000 ***
**Zero Tolerance**	−0.184	0.000 ***	−0.248	0.000 ***	−0.283	0.000 ***
**MLDA**	−0.012	0.006 ***	−0.011	0.010 **	−0.004	0.018 **
**Speed limit**	0.113	0.068 *	0.129	0.072	0.137	0.074
**Open Container**	−0.142	0.000 ***	−0.193	0.000 ***	−0.103	0.025 **
**DUI fine**	−0.034	0.092 *	−0.006	0.332	−0.042	0.033 **
**North West**	−0.330	0.000 ***	−0.321	0.000 ***	−0.344	0.000 ***
**Midwest**	−0.344	0.000 ***	−0.328	0.000 ***	−0.331	0.000 ***
**West**	−0.263	0.000 ***	−0.236	0.000 ***	−0.158	0.000 ***
**Constant**	2.447	0.000 ***	2.633	0.000 ***	2.425	0.000 ***
**Pseudo-R^2^**	0.505		0.571		0.498	
**Obs. number**	1344		1344		1344	

*Notes*: 1. The 25, 50, and 75 percentiles represent the areas with 25th, 50th, and 75th percentiles of the rate of alcohol-related fatality; 2. ARFR, Beer tax, income, unemployment rate, and population density are in natural logarithms; 3. The geographic area “South” is omitted for the comparison base; 4. ***, **, * represent significance levels of 1%, 5%, and 10%, respectively; 5. State-specific time dummies were also included in the regressions while their coefficients are not reported to reduce paper length.

From these analyses, we observed that in the areas with low alcohol-related fatalities, in addition to the increased fatalities caused by economic pressure from unemployment and low per capita income [46], the effects of young drivers on increased alcohol-related fatalities should not be overlooked. In summary, in these areas, alcohol abuse and poor attitudes toward alcohol had a more severe effect on alcohol-related fatalities than poor traffic conditions [6]. Therefore, preventive regulations that are intented to prevent drunk driving were more effective and important than *ex-post* regulations that are intended to penalize drunk driving offenders.

### 4.2. Areas with Medium Alcohol-Related Fatalities

In the areas with medium rates of alcohol-related fatalities, the effects of the speed limit were insignificant, that is, the speed limit in these areas failed to effectively reduce rates of alcohol-related fatalities. Other drinking and driving policies had significant effects on the rates of alcohol-related fatalities in these areas. The beer tax was still the most effective in reducing rates of alcohol-related fatalities. Assuming that other conditions remained constant, when the beer tax increased by 1%, fatalities declined by 0.31%. Additionally, the zero tolerance, open container, and BAC regulations in these areas effectively reduced the rates of alcohol-related fatalities, by 0.25%, 0.19%, and 0.07%, respectively.

In these areas, the number of young drivers had no significant effects on alcohol-related fatalities, indicating that young drivers were not the major cause or focus of alcohol-related fatalities in these areas. Other economic and demographic variables (such as per capita income, unemployment rates, and population density) had significant effects on fatalities at 10% significance level. In particular, per capita income and population density had significant negative correlations with alcohol-related fatalities, that is, when per capita income or population density increased, fatalities declined by 0.016% and 0.165%, respectively. Unemployment rates had a significant positive correlation with fatalities at 10% significance level. Assuming that other conditions remained constant, when unemployment rates increased by 1%, fatalities increased by 0.011%.

From these analyses we observed that in the areas with medium alcohol-related fatalities, traffic conditions should be improved and alcohol abuse and poor attitudes toward alcohol should be discouraged to reduce alcohol-related fatalities. In summary, preventive and ex-post regulations were both significant.

### 4.3. Areas with High Alcohol-Related Fatalities

Most of the included drinking and driving policies all had significant effects in the areas with high alcohol-related fatalities. In particular, the three most effective traffic laws for reducing fatalities were zero tolerance, open container, and BAC for reducing fatalities rates by 0.28%, 0.103%, and 0.101%, respectively. The only traffic law that showed insignificant result was speed limit. In these areas, fewer economic and demographic variables (only per capita income and population density) had significant effects on reducing alcohol-related fatalities, indicating that unemployment rates and the number of young drivers were not major causes of drunk driving in these areas. In summary, improving traffic conditions or creating safe traffic conditions is essential for reducing alcohol-related fatalities in these areas. Additionally, ex-post regulations such as zero tolerance and BAC were relatively more effective than preventive ones.

### 4.4. Comparisons across Quantiles

To test whether all three quantiles were statistically different from each other, Chow tests were performed and presented in Table 3, which indicates that the QR results were significantly different at 5% level for each pair of QR comparison. For systemic comparisons between coefficients across quantiles, differences between coefficients for each variable were computed and the results are presented in Table 3. For the laws that are more effective in the areas with low alcohol-related fatalities, negative numbers appear in the columns of coefficient difference throughout the three pairs of comparison were obtained, which were MLDA and speed limit. Beer tax also worked more effectively in the areas with low alcohol-related fatalities. BAC and zero tolerance, on the other hand, are more effective in the areas with high alcohol-related fatalities. Thus, the areas with different conditions of alcohol-related fatalities should focus on different policies when enforcing the laws. In short, compared with areas that had low fatalities, the effects of preventive regulations for suppressing alcohol-related fatalities had declined in the areas with high fatalities, whereas the effects of ex-post regulations for suppressing fatality rates had increased in general. (The changes of the effects of preventive regulations are as follows: MLDA declined from 0.012% to 0.004%, and the Open Container Law from 0.142% to 0.103%. The changes of the effects of ex-post regulations are as follows: Zero Tolerance increased from 0.184% to 0.283%, BAC (0.08) increased from 0.066% to 0.101%, ALR from 0.058% to 0.065%, and DUI fines from 0.034% to 0.042%.)

**Table 3:** Comparison between coefficients from different quantile regressions.

Difference Between Coefficients						
Variable	25 *vs*. 50 percentile		50 *vs*. 75 percentile		25 *vs*. 75 percentile	
(ARFR)	Difference	*t*-value	Difference	*t*-value	Difference	*t*-value
**Income**	−0.02	0.071 *	0.01	0.076 *	−0.01	0.043 **
**Unemp. rate**	0.02	0.031 **	0.007	0.032 **	0.027	0.008 ***
**Pop. Density**	−0.001	0.097 *	−0.011	0.042 **	−0.012	0.071 *
**Under24**	0.039	0.021 **	0.036	0.976	0.075	0.057 *
**Beer tax**	−0.101	0.071 *	−0.06	0.002 ***	−0.161	0.008 ***
**Belt**	0.013	0.023 **	−0.022	0.047 **	−0.009	0.073 *
**ALR**	−0.004	0.085 *	0.011	0.058 *	0.007	0.078 *
**Bac08**	0.006	0.047 **	0.029	0.094 *	0.035	0.046 *
**Zero Tolerance**	0.064	0.095 *	0.035	0.012 **	0.099	0.023 **
**MLDA**	−0.001	0.057 *	−0.007	0.036 **	−0.008	0.024 **
**Speed limit**	−0.016	0.049 *	−0.008	0.038 **	−0.024	0.017 **
**Open Container**	0.051	0.026 **	−0.09	0.069 *	−0.039	0.053 *
**DUI fine**	−0.028	0.051 *	0.036	0.029 **	0.008	0.072 *
**North West**	−0.009	0.052 *	0.023	0.083 *	0.014	0.045 **
**Midwest**	−0.016	0.066 *	0.003	0.091 *	−0.013	0.057 *
**West**	−0.027	0.043 **	−0.078	0.000 ***	−0.105	0.000 ***
**Chow test**	0.046 **		0.031 **		0.026 **	

*Notes*: 1. Each column presents the difference of coefficients between different quantile regressions; 2. ***, **, * represent significance levels of 1%, 5%, and 10%, respectively; 3. The differences in coefficients of state-specific time dummies and constants are not shown.

## 5. Discussion

It is important for the relevant authorities to gain area-specific understanding of laws when amending them in order to save more lives from drinking and driving. Thus, we used the results from the empirical study on relevant policies to verify the arguments and discourse described above. Comparing the effects of all traffic laws in the three different quantiles, the most effective ones are the same for all three quantiles in the same order—zero tolerance, open container, and BAC. However, some laws are more effective in the areas with high alcohol-related fatalities, some are more effective in the areas with low alcohol-related fatalities, and others may not show consistent patterns across quantiles. In the areas with low alcohol-related fatalities, preventive regulations (beer tax, MLDA, and open container) may be more effective than *ex-post* regulations (such as BAC and zero tolerance), whereas *ex-post* regulations were more effective in areas with high fatalities, with an increase in effectiveness of 0.04% to 0.10% compared with their influence in the areas with low fatalities. Beer tax is most effective for the areas with low rate of alcohol fatalities but zero tolerance is most effective for the areas with high alcohol fatalities. DUI fine laws are effective for the areas with high alcohol fatalities but not so effective for the medium and low rates of alcohol fatalities.

These analyses show that the effectiveness of drinking and driving policies differed in areas with different rates of alcohol-related fatalities. Our results of all the policy effectiveness were statistically significant at 10% level or higher, except for DUI and speed limit in the areas with medium or high rates of alcohol-related fatalities. Even though the results were statistically significant in general, they might not imply social significance given the fact that the effectiveness (the magnitude of coefficients) of the laws was small. The law with greatest impact was zero tolerance, which decreased the rate of alcohol-related fatalities by 0.184%, 0.248%, and 0.283% in the areas with low, medium, and high rates of alcohol-related fatalities, respectively (as shown in Table 2). However, these figures could be translated to 18.82, 25.36, and 28.94 lives saved, respectively, given that total 10,228 people were killed in alcohol-impaired driving crashes in 2012 (Dept of Transportation 2012). While this study did not intend to address the issue of social significance (To determine whether the results are socially significant, which can be referred to changes on measures that are *important* to society, some cut off points or thresholds need to be carefully defined [47,48], which is beyond the scope of this study.) and the implementation of each traffic law did not seem to save many lives, it is believed that each life counts and is of great importance to their family. Therefore, it is crucial for the relevant authorities to gain better understanding of traffic laws. When deciding on methods by which to lower alcohol-related fatalities, the U.S. states should consider the characteristics of drunk driving in their areas to effectively reduce fatality rates.

## 6. Conclusions

The statistics from the FARS of the NHTSA show that approximately 30,000 people were killed or injured in car crashes in the U.S. in 2009. Forty percent of these crashes occurred during weekends (approximately 12,000 casualties), possibly because people consume excessive quantities of alcohol at social engagements on weekends, causing severe alcohol-related crashes [37]. This indicates that drunk driving remains a severe social problem in the U.S. that motivates scholars and experts to identify factors that can reduce alcohol-related fatalities.

In this study, we used the alcohol-related fatalities per 100,000 people in the U.S. states between 1980 and 2009 for our analysis. The data show the following phenomena: (1) consistency: areas with high rates of alcohol-related fatalities in the 1980s remained so in 2009; and (2) regionality: areas with higher rates of alcohol-related fatalities were situated in the west and south, whereas areas with lower alcohol-related fatalities were situated in the northeast. These characteristics led us to question if drinking and driving policies had the same effects in areas with different rates of alcohol-related fatalities. Therefore, we used the QR method to discuss the effectiveness of various drinking and driving policies for different quantiles of alcohol-related fatalities.

The results from the empirical study show demographic factors such as income, unemployment rates, young driver ratio, and population density were all significant in areas with low rates of alcohol-related fatalities; while only income and population density were significant in areas with high rates of alcohol-related fatalities. Considering the numbers of coefficients, we also find that lower beer tax and declined economic conditions (such as decreased income or increased unemployment) are correlated with higher rate of alcohol-related fatalities with impact greater in areas with low alcohol-related fatalities than in high fatality areas. Additionally, increased numbers of young drivers in areas with low rates of alcohol-related fatalities result in increased fatalities, whereas they did not significantly affect the fatalities in the areas with higher rates of alcohol-related fatalities. This implies that in areas with low alcohol-related fatalities (as compared to high fatality areas), drinking habits and attitudes may be restrained more easily by stricter drinking and driving policies and these areas are influenced to a greater extent by economic and demographic conditions. On the other hand, drinking habits and attitudes may not be easily changed in the areas with high alcohol-related fatalities; *ex-post* regulations are thus important for discouraging drinking people driving on the road. As a result, *ex-post* regulations are more important in the areas with high fatalities whereas preventive regulations are intended to prevent alcohol abuse and thus decrease alcohol-related fatalities in the areas with low fatalities.

## Appendix

**Table A1:** Abbreviation of the states in the USA (by alphabetic order).

State	Abbreviation	State	Abbreviation
Alabama	AL	Montana	MT
Alaska	AK	Nebraska	NE
Arizona	AZ	Nevada	NV
Arkansas	AR	New Hampshire	NH
California	CA	New Jersey	NJ
Colorado	CO	New Mexico	NM
Connecticut	CT	New York	NY
Delaware	DE	North Carolina	NC
Florida	FL	North Dakota	ND
Georgia	GA	Ohio	OH
Hawaii	HI	Oklahoma	OK
Idaho	ID	Oregon	OR
Illinois	IL	Pennsylvania	PA
Indiana	IN	Rhode Island	RI
Iowa	IA	South Carolina	SC
Kansas	KS	South Dakota	SD
Kentucky	KY	Tennessee	TN
Louisiana	LA	Texas	TX
Maine	ME	Utah	UT
Maryland	MD	Virginia	VA
Massachusetts	MA	Vermont	VT
Michigan	MI	Washington	WA
Minnesota	MN	West Virginia	WV
Mississippi	MS	Wisconsin	WI
Missouri	MO	Wyoming	WY
